# Congenital Protein C Deficiency Presenting as Neonatal Purpura Fulminans: A Report of Two Cases

**DOI:** 10.7759/cureus.103740

**Published:** 2026-02-16

**Authors:** Rekha Thaddanee, Sandeep Tilwani, Taral Kesharani, Jui Shah, Nausheen Sheikh

**Affiliations:** 1 Pediatrics, Gujarat Adani Institute of Medical Sciences, Bhuj, IND; 2 Dermatology, Gujarat Adani Institute of Medical Sciences, Bhuj, IND

**Keywords:** antithrombin iii deficiency, hereditary protein s deficiency, neonatal purpura fulminans, protein c and s deficiency, severe protein c deficiency

## Abstract

Purpura fulminans (PF) is a rare, life-threatening thrombotic disorder characterized by progressive cutaneous hemorrhagic necrosis and disseminated intravascular coagulation (DIC). Neonatal PF may result from homozygous or compound heterozygous deficiencies in natural anticoagulants, such as protein C, protein S, or antithrombin III, or secondary to sepsis. Laboratory findings typically show consumptive coagulopathy with thrombocytopenia, prolonged prothrombin time (PT), activated partial thromboplastin time (aPTT), elevated international normalized ratio (INR), low fibrinogen, and high D-dimer levels. This report describes two full-term male neonates born to consanguineous parents who developed early-onset PF due to severe hereditary protein-C deficiency (activity 4-8%, below the normal neonatal range of 25-40 IU/dL or 14-42%).​ Both exhibited rapidly progressive ecchymotic lesions leading to necrosis and eschar, and despite fresh frozen plasma (FFP) transfusions, heparin, and supportive care, they succumbed to DIC and sepsis on 13 and 22 days of life, respectively. These cases contribute meaningfully to the existing literature and reinforce the importance of genetic counseling, prompt diagnosis, and strengthening neonatal critical care resources.

## Introduction

Purpura fulminans (PF) is an acute thrombotic disorder involving microvascular thrombosis, leading to hemorrhagic skin necrosis, perivascular bleeding, and disseminated intravascular coagulation (DIC). It is extremely uncommon for congenital protein C deficiency to show up as PF in newborns - only one in four million births [1,2]. Acquired neonatal PF can be due to severe neonatal sepsis caused by organisms such as group B streptococcus and *Staphylococcus aureus*. Protein C functions as a major anticoagulant protein by inactivating factors Va and VIIIa that are key drivers of the coagulation cascade. When protein C levels fall drastically below normal, excessive clotting can damage blood vessels and lead to widespread microthrombi formation. In this condition, protein C levels or activity are found to be lower than the reference range, with normal values in full-term infants being at least 25 IU/dL [3,4].

Clinically, it manifests as non-blanchable purpura or ecchymosis surrounded by erythematous halos, rapidly evolving into bullae, necrosis, and eschar. Severe homozygous protein C deficiency can be incompatible with life without anticoagulation and replacement therapy with fresh frozen plasma (FFP) or protein C concentrates [5]. We are reporting two newborn cases of PF due to protein C deficiency.

## Case presentation

Case 1

**Figure 1:**
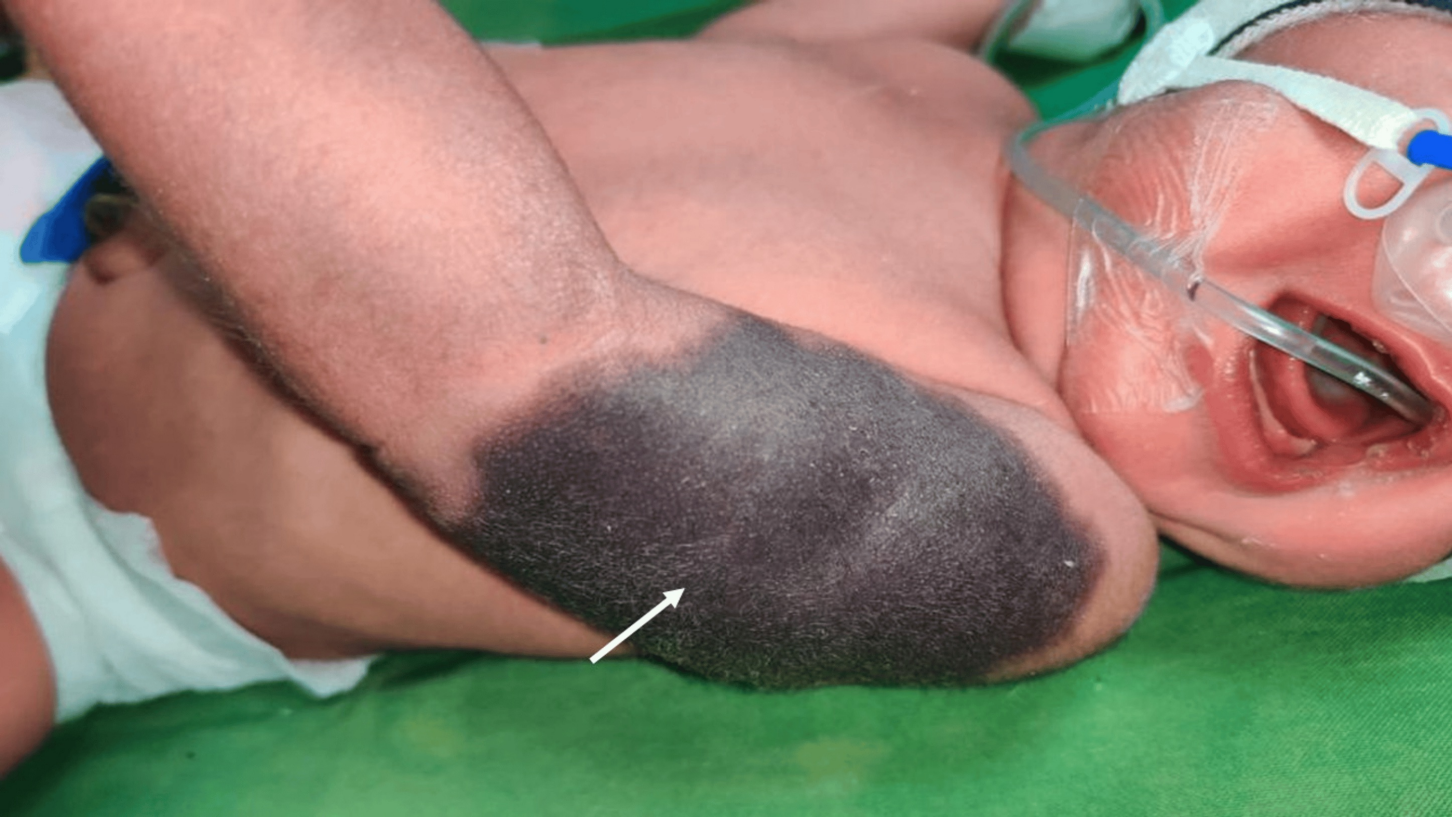
Ecchymotic patch over the left arm (white arrow)

A full-term male neonate with normal birth weight (3.18 kg) was admitted to the Neonatal Intensive Care Unit (NICU) for mild respiratory distress. Perinatal history revealed a second-degree consanguineous marriage. The mother was gravida 4, para 3, with three living children and one prior abortion (G4P3L3A1). She had not received any antenatal care during the current pregnancy. At 12 hours of life, the baby had a well-defined ecchymotic patch over the left arm (Figure 1). 

On examination, his heart rate was 150 beats per minute, respiratory rate was 58 breaths per minute, temperature was 98.8 °F, and capillary refill time was < 2 seconds. There was no history of trauma or clinical evidence of sepsis. Skin lesion over the left arm progressively increased in size on the third day of life.

Baseline investigations revealed hemoglobin (Hb) of 15 g/dL, total leukocyte count (TLC) of 15,000 cells/mm³, and platelet count (PC) of 67,000/µL, indicating thrombocytopenia. Coagulation profile showed a markedly prolonged prothrombin time (PT) of 56 seconds, a mildly prolonged activated partial thromboplastin time (aPTT) of 38 seconds, and an elevated INR of 6.6. Fibrinogen levels were significantly reduced (<40 mg/dL), and D-dimer levels were elevated (4,000 ng/mL). All blood investigations were sent before administering the FFP. Blood culture was sterile, and arterial and venous Doppler studies were normal. Thus, a provisional diagnosis of neonatal PF was made.

**Figure 2:**
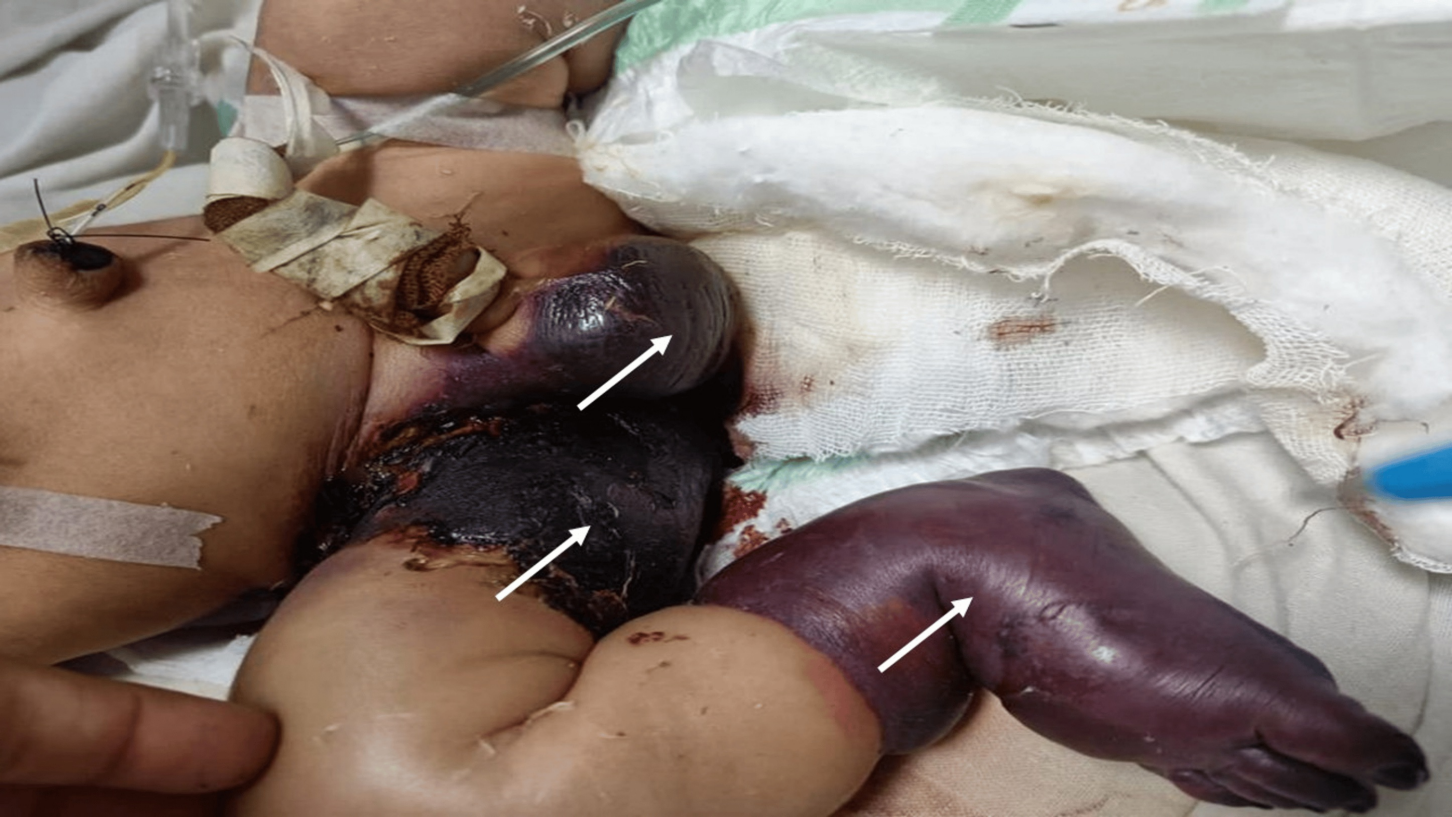
Ecchymotic lesions with necrotic changes over the left hand, right scrotum, thigh, and lower extremity (white arrows)

Despite aggressive therapy, by the ninth day of life, similar ecchymotic lesions appeared and rapidly progressed to necrosis, culminating in black eschar formation over the genital and limb regions (Figure 2). 

**Figure 3:**
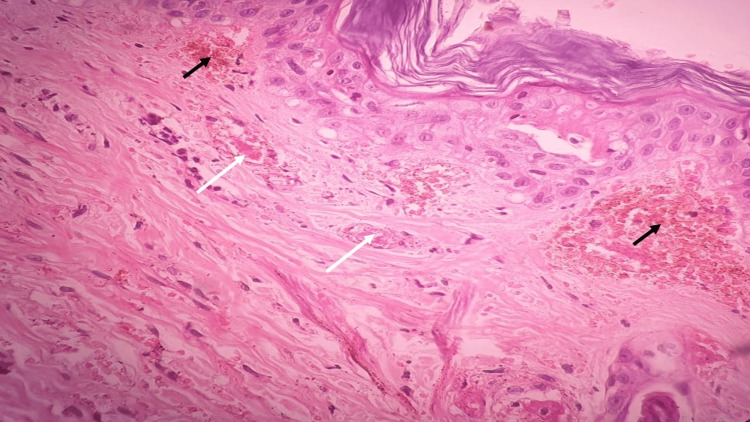
Hematoxylin and eosin staining (x 40) showed intravascular thrombus formation (white arrow) and hemorrhagic areas (black arrow)

Skin biopsy from the lesion on the right thigh revealed intravascular thrombus formation within small to medium-sized vessels along with extensive hemorrhagic necrosis, confirming the diagnosis of neonatal purpura fulminans (Figure 3).

Case 2

**Figure 4:**
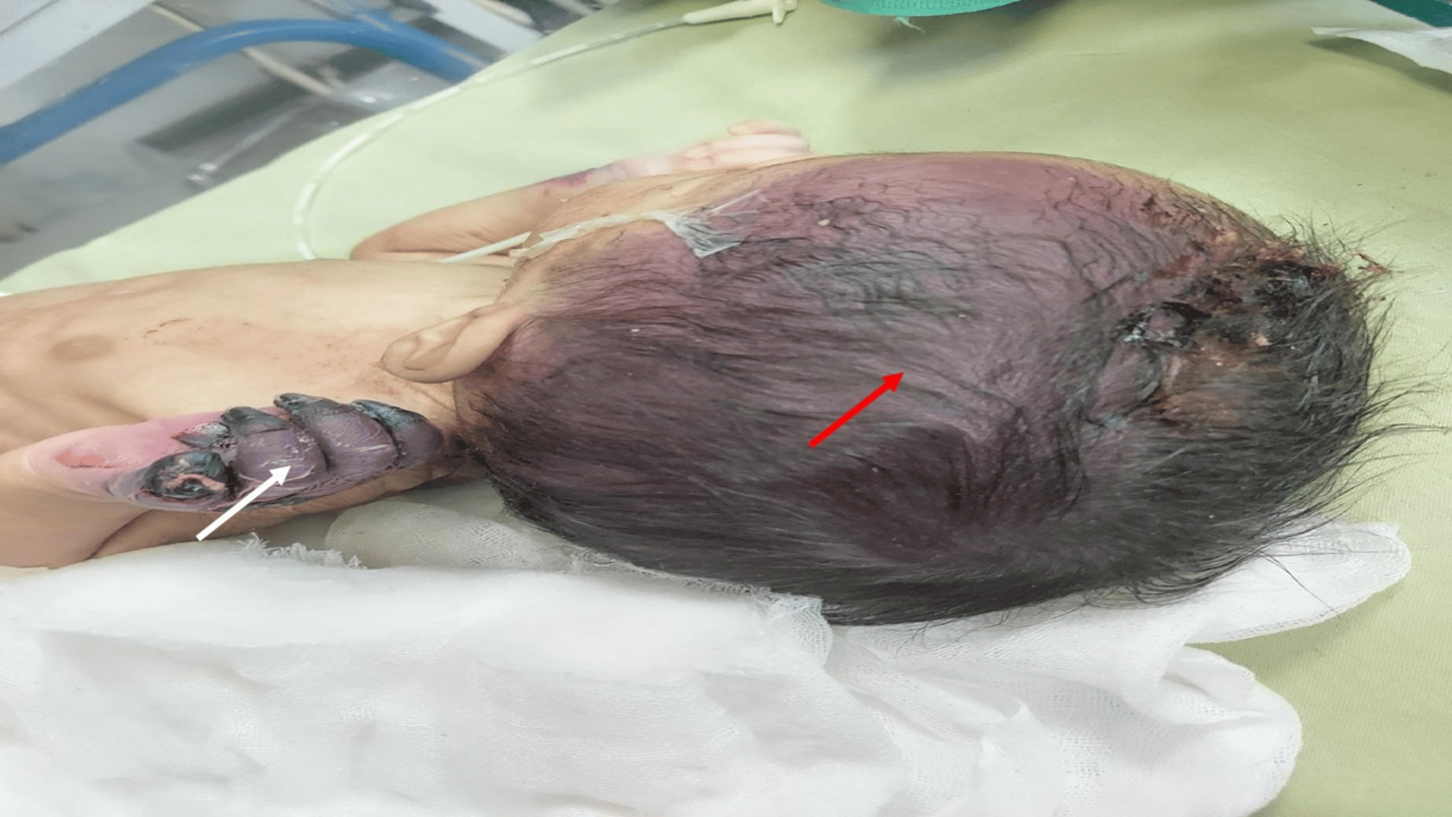
Ecchymotic patch over the left hand (white arrow) and scalp (red arrow) with necrotic changes and bulla formation

A full-term male neonate with low birth weight (2.15 kg) delivered by lower segment cesarean section (LSCS) was referred for multiple ecchymotic patches over the scalp, trunk, and upper limbs appearing within 24 hours of life. The lesions increased in size and number (Figure 4). 

As in Case 1, a history of second-degree consanguinity was present. On examination, all vitals were normal. Investigations revealed Hb of 11.2 g/dL, TLC of 16,740 cells/mm³, PC of 42,000/µL, PT of 28.8 seconds, aPTT of 49.9 seconds, INR of 2.8, fibrinogen levels of <40 mg/dL, and D-dimer levels of > 8,000 ng/mL (Table 1).

**Table 1:** Blood parameters of both cases as compared to the normal neonatal range Hb: Hemoglobin, TLC: Total Leukocyte Count, PC: Platelets Count, PT: Prothrombin Time (seconds), aPTT: Activated Partial Thromboplastin Time (seconds), INR: International Normalized Ratio Normal Neonatal Range [6]

Blood Parameter	Case 1	Case 2	Normal Neonatal Range
Hb (g/dL)	15	11.2	14-18​
TLC (cells/mm³)	15,000	16,740	9000-30,000
PC (×10³/µL)	67	42	150-450
PT (seconds)	56	28.8	11-15
aPTT (seconds)	38	49.9	29-35
INR	6.6	2.8	< 1.2
Fibrinogen (mg/dL)	< 40	< 40	150-400
D-dimer (ng/mL)	4000	> 8000	500-2500
Protein C Activity (%)	8.0	4	14-42
Protein S Activity (%)	56	51	33-67
Antithrombin III (%)	48	42	39-97

**Figure 5:**
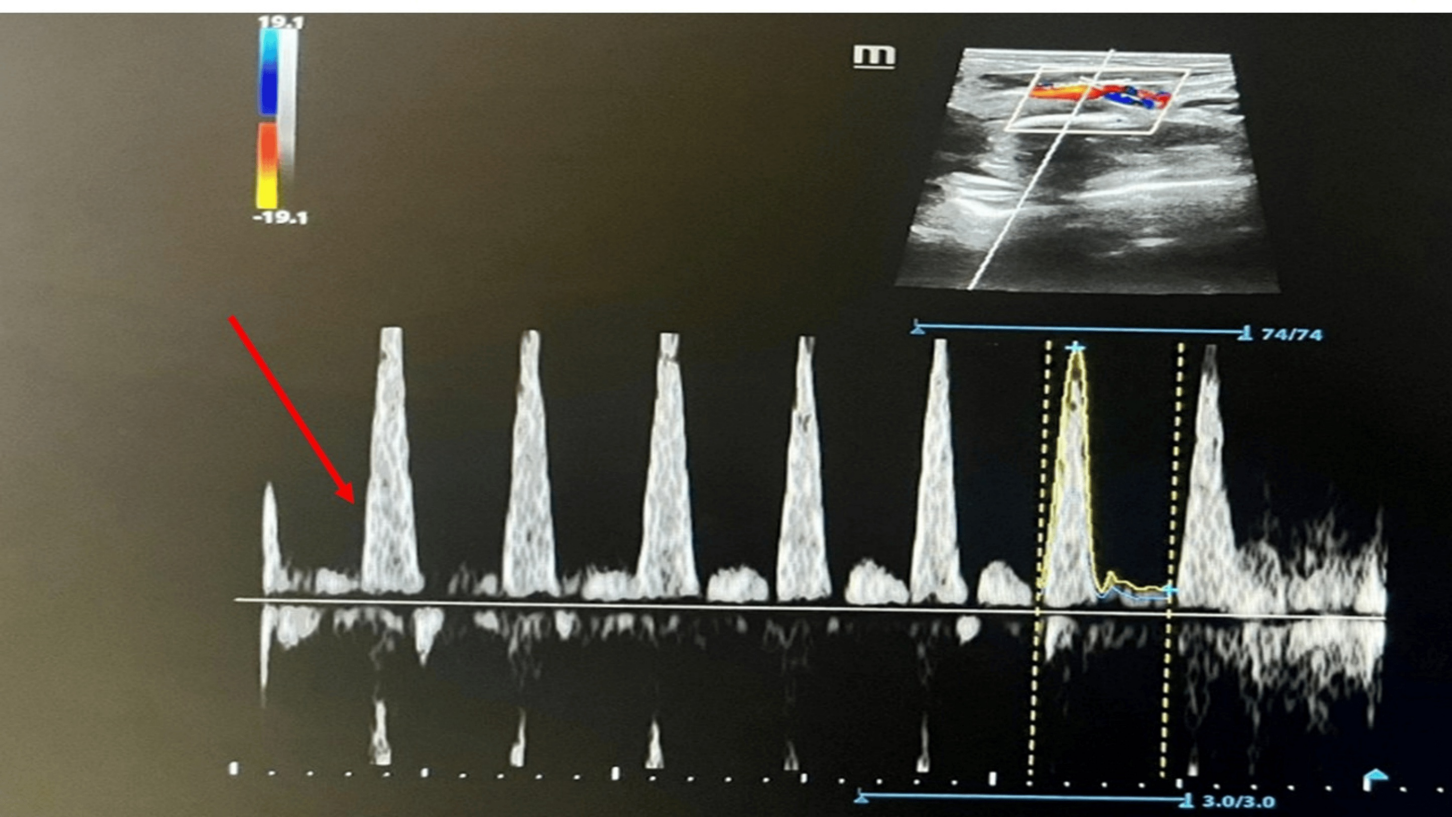
Right femoral artery Doppler showing normal triphasic waveforms (red arrow)

Blood culture was sterile. Doppler studies were normal (Figure 5). Thus, a provisional diagnosis of PF was made. 

For confirmation, protein C, protein S, or antithrombin III levels were sent in both cases. These investigations revealed markedly reduced protein C activity (8.0% in Case 1 and 4% in Case 2), with normal protein S and antithrombin III levels in both cases, indicative of hereditary protein C deficiency. Genetic testing could not be performed due to prior plasma transfusions.

Both babies were managed with oxygen support, intravenous fluid, nasogastric feeds, antibiotics, multiple transfusions of FFP (10 mL/kg every 12 hours), and continuous intravenous unfractionated heparin infusion initiated at 28 U/kg/kg/h. Protein C concentrate was not available. Despite all supportive measures, progressive skin necrosis, DIC, and sepsis ensued, and both cases succumbed on the 22nd and 13th day of life, respectively.

## Discussion

PF was first described in 1884. Hereditary neonatal form due to severe protein C deficiency is extremely rare, with an estimated frequency of one per 1,000,000 live births [3]. Inherited protein C deficiency follows an autosomal dominant (single altered copy of the *PROC *gene - milder form) or autosomal recessive pattern (both altered copies of the *PROC *gene - severe form), although spontaneous mutations have been reported. Over 160 mutations in the *PROC *gene on chromosome 2q14.3 have been identified [4]. Two major types are recognized: type I deficiency with reduced protein C antigen and activity levels and type II deficiency with normal antigen levels but reduced activity levels [7].

Individuals carrying a single altered gene copy (heterozygotes) typically show reduced protein C levels and activity, predisposing them to recurrent venous thromboembolism, often developing later in life. Some may remain asymptomatic. In contrast, babies born with two abnormal alleles (homozygous or compound heterozygous cases) suffer from much more pronounced symptoms, including severe clotting disorders, such as PF and disseminated intravascular coagulation, within the neonatal period due to extremely low protein C activity [8]. In healthy term newborns, plasma protein C activity is typically around 25-40 IU/dL or about 14-42% (5th-95th percentile) of adult levels [9]. In both cases, severe congenital protein C deficiency (4-8%) was present.

Prompt recognition and aggressive management are essential. The cornerstone of therapy is replacing deficient protein C activity, ideally with protein C concentrate, or if unavailable, with FFP. Protein C concentrate is preferred as it corrects the deficiency without causing volume overload. FFP (10-15 mL/kg every 8-12 hours) is the alternative mainstay when concentrate is not available. Despite aggressive early intervention, outcomes remain poor in patients lacking access to specific protein C replacement therapy, as seen in our unfortunate cases.

Adjunctive anticoagulation with unfractionated or low-molecular-weight heparin can help prevent new thrombus formation. Supportive care involves maintaining adequate perfusion, infection control, and managing DIC. For severe or recurrent cases, long-term prophylaxis with protein C concentrate, oral anticoagulants, or liver transplantation (in cases requiring definitive cure) may be considered [10].

Our cases also emphasize the importance of considering familial and genetic factors, particularly consanguinity, as such relationships increase the probability of inheriting autosomal recessive disorders such as protein C deficiency. Genetic counseling and screening in families at risk can facilitate early diagnosis and preventive strategies, including prenatal diagnosis and rapid postnatal intervention in subsequent pregnancies.

## Conclusions

Congenital protein C deficiency, causing neonatal PF, is an extremely rare but devastating condition. Early onset of progressive purpura, thrombocytopenia, and coagulopathy in the absence of infection should immediately raise suspicion of an inherited thrombophilia. Timely diagnosis through coagulation studies and specific assays for protein C, protein S, and antithrombin III is crucial. Management requires prompt replacement therapy with protein C concentrate or, when unavailable, repeated FFP transfusions alongside anticoagulation and supportive care.
